# Heat shock protein 90 targeting therapy: state of the art and future perspective

**DOI:** 10.17179/excli2014-586

**Published:** 2015-01-06

**Authors:** Manabu Tatokoro, Fumitaka Koga, Soichiro Yoshida, Kazunori Kihara

**Affiliations:** 1 Department of Urology, Tokyo Medical and Dental University Graduate School, Tokyo, Japan; 2 Department of Urology, Tokyo Metropolitan Cancer and Infectious diseases Center Komagome Hospital, Tokyo, Japan

**Keywords:** Hsp90 inhibitor, cancer, clinical trial, bladder cancer

## Abstract

Heat shock protein 90 (Hsp90) is an ATP-dependent molecular chaperone that plays a role in stabilizing and activating more than 200 client proteins. It is required for the stability and function of numerous oncogenic signaling proteins that determine the hallmarks of cancer. Since the initial discovery of the first Hsp90 inhibitor in the 1970s, multiple phase II and III clinical trials of several Hsp90 inhibitors have been undertaken. This review provides an overview of the current status on clinical trials of Hsp90 inhibitors and future perspectives on novel anticancer strategies using Hsp90 inhibitors.

## Introduction

Heat shock protein 90 (Hsp90) is one of the most abundant proteins in eukaryotes, comprising as much as 1 to 2 % of the total cellular protein content under non-stressed conditions, and increasing approximately twofold during environmental stress (Buchner, 1999[2]; Welch and Feramisco, 1982[54]; Whitesell and Lindquist, 2005[56]). In human cells, Hsp90 can be found in the cytosol, nucleoplasm, endoplasmic reticulum and mitochondria (Chen et al., 2005[3]). Beyond Hsp90's essential role in maintaining normal tissue homeostasis, it is an ATP-dependent molecular chaperone that plays a role in stabilizing and activating more than 200 client proteins, many of which are essential for constitutive cell signaling and adaptive responses to stress (Neckers, 2007[33]; Trepel et al., 2010[52], Whitesell and Lindquist; 2005[56]). The client proteins include oncogenic tyrosine kinase v-Src, mutated oncogene Bcr/Abl, receptor tyrosine kinase of the erbB family and c-MET, and serine/threonine kinase Raf-1 as well as transcription factors such as hypoxia-inducible factor-1α, tumor suppressor p53 protein, and steroid receptors (Koga et al., 2009[22]; Tsutsumi et al., 2009[53]).

## Cancer and Hsp90

Cancer is a disease of genetic instability (Neckers, 2007[33]). For example, in colorectal cancers, 5 to 10 genetic alterations appear necessary for the generation of the malignant phenotype, with the classical example of a multistep progression pathway for sporadic carcinoma; however, over 10,000 genomic events per carcinoma have been found at the time of diagnosis (Stoler et al., 1999[47]). The genetic instability allows a cancer cell to acquire the eight hallmarks proposed by Hanahan and Weinberg (2000[13], 2011[12]). These are (i) self-sufficiency in growth signals; (ii) insensitivity to anti-growth signals; (iii) evading apoptosis; (iv) sustained angiogenesis; (v) tissue invasion and metastasis; (vi) limitless replicative potential; (vii) reprogramming of energy metabolism; and (viii) evading immune destruction. These hallmarks reflect genetic alterations in multiple safeguard genes responsible for the regulation and tight coordination of diverse processes, such as cell survival, proliferation, growth, differentiation, and motility (Sidera and Patsavoudi, 2014[44]). This genetic plasticity allows cancer cells to escape the precise molecular targeting of a single signaling node or pathway, making them ultimately non-responsive to molecularly targeted therapeutics (Neckers, 2007[33]). Each cancer chemotherapeutic has targeted proteins associated with multiple hallmarks, though none have been able to simultaneously affect all of them (Sidera and Patsavoudi, 2014[44]).

Hsp90 consists of a central node in signaling networks and plays a pivotal role in the acquisition and maintenance of each of these capabilities (Neckers, 2007[33]). Therefore, inhibition of Hsp90 leads to the degradation of these oncogenic clients and abrogates the six hallmarks of a cancer cell simultaneously. Therefore, targeting Hsp90 appears to be a reasonable anticancer strategy. 

## Hsp90 Inhibitors

Hsp90 inhibitors bind to Hsp90 and inhibit Hsp90 function by competing with ATP binding, thereby freezing the chaperone cycle, which in turn decreases the affinity of Hsp90 for client proteins and leads to proteasome-mediated client protein degradation (Tsutsumi et al., 2009[53]). Although Hsp90 is highly expressed in most cells, Hsp90 inhibitors kill cancer cells selectively compared to normal cells (Kamal et al., 2003[20]). This therapeutic selectivity results from the activated, high-affinity chaperone of Hsp90 in tumors (Kamal et al., 2003[20]). Based on the above, Hsp90 has emerged as an exciting and promising new target for the development of new antineoplastic agents for a variety of human cancers in the last two decades.

In 1970, the benzoquinone ansamycin antibiotics were first isolated (DeBoer et al., 1970[5]). These include geldanamycin (GA), which was found in a search for compounds able to revert the transformed phenotype of v-src transformed 3T3 cells (Whitesell et al., 1992[58]). However, an *in vitro* kinase assay revealed that GA neither directly interacts with Src nor inhibits its phosphorylating activity. Consequently, Hsp90 was identified as the direct target of GA (Whitesell et al., 1994[57]). GA was shown to mimic the structure adopted by ATP in the N-terminal nucleotide-binding pocket of Hsp90, thus leading to selective inhibition of ATP binding and hydrolysis and, in turn, to the depletion of oncogenic Hsp90 clients (Supko et al., 1995[48]). GA alters chaperone function and drives the degradation of many Hsp90 client proteins by stimulating Hsp90-mediated presentation to the ubiquitin-proteasome machinery. Consequently, the client proteins cannot attain their active conformation and are degraded by the proteasome (Mimnaugh et al., 1996[28]). Although GA is broadly cytotoxic *in vitro*, its poor solubility and intolerable liver toxicity *in vivo* precluded clinical trials (Supko et al., 1995[48]). In the last decade, there has been a considerable increase in the discovery of Hsp90 inhibitors, progressing from first-generation derivatives of natural products to second-generation fully synthetic small molecules (Neckers, 2007[33]; Sidera and Patsavoudi, 2014[44]). Less toxic agents than GA, namely, 17-AAG (17-Allylamino-17-demethoxy- geldanamycin) and 17-DMAG (17-dimethylaminoethylamino-17-demethoxy-geldanamycin), have proceeded to clinical trials. 

### 17-AAG (Tanespimycin)

17-AAG was the first Hsp90 inhibitor to enter clinical trials. *In vitro* and *in vivo*, it has shown antitumor activity in various preclinical models, such as colon, breast, ovarian, and melanoma tumors (Neckers, 2007[33]; Saif et al., 2013[38]). As 17-AAG is not water-soluble and requires a diluent, including egg phospholipid and 4 % DMSO, hypersensitivity reactions were observed in the phase I trial (Whitesell and Lin, 2012[55]). Taken together, several phase II studies of single agent 17-AAG have been performed since 1999 (Gartner et al., 2012[10]; Neckers, 2007[33]; Saif et al., 2013[38]). However, given the lack of response and apparent toxicity, including fatigue, nausea, vomiting, diarrhea, and transaminase elevations, phase II studies in patients with metastatic breast cancer and metastatic melanoma were terminated early (Gartner et al., 2012[10]; Pacey et al., 2012[35]).

### 17-DMAG (Alvespimycin) and IPI-504 (Retaspimycin)

To overcome the formulation issues with 17-AAG, 17-DMAG (Alvespimycin) and IPI-504 (Retaspimycin) was developed as its water-soluble analog. Alvespimycin is associated with a longer plasma half-life, greater oral bioavailability, and less extensive metabolism (Jhaveri et al., 2012[19]). In preclinical studies, it produced superior antitumor activity and lower toxicity compared with 17-AAG (Eiseman et al., 2005[7]; Jhaveri et al., 2012[19]). A phase I trial of 17-DMAG for advanced solid tumors revealed clinical activity in castration-refractory prostate cancer (complete response), melanoma (partial response), renal cancer, and chondrosarcoma (stable disease) (Pacey et al., 2011[36]). In a phase I trial of 17-DMAG for acute myeloid leukemia (AML), the drug was well tolerated and anti-leukemia activity was observed in 3 of 17 evaluable patients (Lancet et al., 2010[26]). A phase II clinical trial of intravenous 17-DMAG for HER2-positive breast cancer was terminated for unknown reasons (Squibb, 2011[46]).

IPI-504 has reached phase III clinical trials. In a randomized, phase III trial of IPI-504 conducted in patients with metastatic and/or unresectable gastrointestinal stromal tumors (GIST), the trial was terminated early due to the occurrence of four on-treatment deaths in the IPI-504 arm. These deaths were considered drug-related and included renal failure, liver failure, metabolic acidosis, and cardiopulmonary arrest (Demetri et al., 2010[6]). In contrast, in some phase II studies, including patients with non-small cell lung cancer (NSCLC) (Sequist et al., 2010[40]) and HER2-positive breast cancer (Modi et al., 2013[30]), IPI-504 had an acceptable safety profile, with infrequent transaminase elevations. 

### Second- and third-generation HSP90 inhibitors

The impressive growth in interest in Hsp90 is evident in both the academic and patent literature and resulted in the discovery and pre-clinical testing of an array of new synthetic inhibitors. Seventeen agents have undergone clinical trials and nine remain under clinical investigation (Neckers and Trepel, 2014[34]). Although no Hsp90-targeting agents have yet achieved an approved indication in the treatment of any cancer, several structurally distinct Hsp90 inhibitors are currently being evaluated for anticancer activity in several phase III clinical trials. These new agents share the ability to bind the N-terminal ATPase site of Hsp90 with higher affinity than the natural nucleotides and prevent the chaperone from cycling between its ADP- and ATP-bound conformations (Whitesell and Lin, 2012[55]). Currently, Hsp90 inhibitors are being evaluated in 52 clinical trials (National Cancer Institute, 2014[31]). AUY922 (Novartis) and STA-9090 (ganetespib, Synta) are furthest in development (Whitesell and Lin, 2012[55]). 

Currently, AUY922 is being evaluated in 13 clinical trials (National Cancer Institute, 2014[31]), including nine phase II trials in patients with NSCLC, gastrointestinal stromal tumor (GIST), and metastatic pancreatic cancer (Table 1(Tab. 1)). Common adverse effects of AUY922 have included diarrhea, nausea, fatigue, vomiting, and ocular toxicities (Sessa et al., 2009[41]). In a phase II trial of AUY922 monotherapy in patients with advanced NSCLC, preliminary clinical activity was seen with partial responses in 13 % (Garon et al., 2012[9]). Numerous phase II monotherapy trials are underway across a variety of malignancies (Whitesell and Lin, 2012[55]).

STA-9090 is an investigational small molecule inhibitor of Hsp90 that has favorable pharmacologic properties that distinguish the compound from other first- and second-generation Hsp90 inhibitors in terms of potency, safety, and tolerability (He et al., 2014[14]). This agent displayed a 20-fold superior potency to 17-AAG in a panel of 57 transformed cell lines of both hematologic and solid tumors (Ying et al., 2012[60]). It has reached phase III clinical trials and 12 clinical trials are currently underway (National Cancer Institute 2014[31]) (Table 2(Tab. 2)). In a multicenter phase II study of STA-9090 monotherapy in patients with advanced NSCLC, durable objective responses and disease stabilization occurred in the majority of patients with disease harboring ALK gene rearrangements that were crizotinib-naïve (Socinski et al., 2013[45]). The most common side effects were diarrhea, fatigue, nausea, and anorexia, which have been manageable with standard care. It is notable that, in contrast to AUY922, no ocular toxicity has been reported for STA-9090. The clinical activity of monotherapy was observed in heavily pretreated NSCLC, breast cancer, gastric cancer, melanoma, and colon cancer (Whitesell and Lin, 2012[55]).

## Combination Activity of Hsp90 Inhibitors

Single agent activity for Hsp90 inhibitors has been disappointingly modest against heavily pretreated cancers in clinical trials that have been reported to date (Trepel et al., 2010[52]; Whitesell and Lin, 2012[55]). Whitesell et al. (2012[55]) suggested this issue might be intrinsic to the target itself and that Hsp90 inhibition could serve as a platform for the assembly of specific multi-drug chemotherapeutic regimens that will more effectively control disparate cancers. Preclinical data from various types of *in* *vitro* and *in vivo* cancer models suggest that Hsp90 inhibitors have the ability to enhance the activity of other anticancer strategies, including chemotherapy, kinase inhibitors, and radiation therapy, to achieve synergistic or additive antitumor effects, and to potentially overcome drug resistance (Jhaveri et al., 2014[18]). This has formed the basis for rational combination trials of Hsp90 inhibitors with other cancer therapeutic agents.

### Chemotherapy

Combinations with several classes of cytotoxic chemotherapeutic agents have now reached clinical trial. Docetaxel has been combined with STA-9090 and IPI-504 in a clinical trial of NSCLC. A phase III trial of STA-9090 in combination with docetaxel versus docetaxel alone in patients with advanced NSCLC is ongoing (Goss et al., 2012[11]). Recently, Synta Pharmaceuticals Corp. announced the results from the final analysis of this trial. For chemo-sensitive patients in particular, the improvements in progression-free survival and overall survival with STA-9090 and docetaxel were encouraging (Synta Pharmaceuticals Corp., 2014[50]). The phase II randomized trial that evaluated the efficacy and safety of IPI-504 plus docetaxel compared to placebo plus docetaxel in 226 patients with NSCLC was completed. Although the safety profile of IPI-504 plus docetaxel was comparable to docetaxel and placebo, IPI-504 did not meet its pre-specified efficacy endpoints for demonstrating an improvement in overall survival (Infinity Pharmaceuticals, 2013[16]).

### Kinase inhibitors

Hsp90 inhibition may also represent an effective strategy to overcome or delay the development of tyrosine kinase inhibitor resistance (Neckers and Trepel, 2014[34]). Preclinical and clinical examples of crizotinib-resistant ALK mutations responding to Hsp90 inhibition have also been reported (Socinski et al., 2013[45]). Furthermore, synergistic growth inhibition in MET-driven tumor models upon combining an Hsp90 inhibitor and a kinase inhibitor targeting this Hsp90-dependent kinase was recently reported (Miyajima et al., 2013[29]). A phase II clinical trial evaluated the safety and efficacy of crizotinib and STA-9090 in ALK-positive lung cancers, and evaluation of the clinical benefit of STA-9090 in combination with sirolimus for patients with unresectable or metastatic malignant peripheral nerve sheath tumor is ongoing (National Cancer Institute, 2014[31]). 

### Radiation therapy

Radiation therapy is a well-established standard treatment option for localized and locally advanced cancer. An approach to augment the efficacy of radiation therapy without simultaneously increasing the risk to normal tissues is biologic escalation of the radiation dose to the tumor through the use of tumor-specific radiosensitizing agents (Gandhi et al., 2013[8]). Targeting Hsp90 is a radiosensitizing approach for cancer cells in which Hsp90 is overexpressed compared to normal cells. Hsp90 inhibition offers the possibility of radiosensitization through broad downregulation of multiple critical radioresistance pathways whose components are members of the Hsp90 clientele, such as signal transduction pathways (PI3K-Akt-mTOR) and DNA damage response pathways (ATR/Chk1) (Gandhi et al., 2013[8]). 17-AAG has been validated as a potential therapeutic agent that can be used at clinically relevant doses to enhance cancer cell sensitivity to radiation. 17-AAG has been reported to potentiate both the *in* *vitro* and *vivo* radiation response of cervical carcinoma cells (Bisht et al., 2003[1]). STA-9090 acts as a radiosensitizer to potentiate the effects of low-dose radiation *in* *vitro *(He et al., 2014[14]). At the molecular level, it was found that combined Hsp90 inhibition and radiation impacted several overlapping pathways that led to cell cycle dysregulation, diminished DNA repair capacity, and enhanced apoptosis. Currently, a phase I clinical trial of STA-9090 given together with capecitabine and radiation in patients with locally advanced rectal cancer is ongoing (National Cancer Institute 2014[31]).

## Hsp90 Inhibitors in Bladder Cancer

For a decade, we have investigated the possibility of the clinical application of HSP90 inhibitors, with a particular focus on bladder cancers. Similar to other malignancies, the single agent activity of Hsp90 inhibitors has been disappointingly modest against bladder cancers and there are no active clinical trials.

### Combination of Hsp90 inhibitor in chemoradiotherapy-based bladder-sparing treatment for muscle-invasive bladder cancer

Bladder cancer is the fifth most common cancer in the US, with 74,690 new patients and 15,580 deaths being estimated in 2014 (National Cancer Institute 2014[32]). Muscle-invasive bladder cancer (MIBC) accounts for one-third of all bladder cancer cases (Lee and Droller, 2000[27]). Radical cystectomy with urinary diversion, the reference standard treatment for MIBC, is associated with high complication rates and compromises quality of life (QOL) as a result of long-term effects on urinary, gastrointestinal and sexual function, and changes in body image. As a society ages, the number of elderly patients unfit for radical cystectomy as a result of comorbidity will increase, and the demand for bladder-sparing approaches for muscle-invasive bladder cancer will thus inevitably increase (Koga and Kihara, 2012[21]). To overcome these issues, bladder-sparing approaches combined with various modalities have been investigated (Housset et al., 1993[15]; Koga et al., 2012[23]; Shipley et al., 2002[42]). Above all, bladder-sparing approaches incorporating chemoradiotherapy (CRT) improves QOL while not compromising survival outcomes in MIBC patients. In most bladder-sparing protocols, complete response to induction CRT is a prerequisite for bladder preservation, also indicating favorable oncological outcomes (Koga et al., 2012[23], 2008[24]; Rodel et al., 2002[37]: Shipley et al., 1987[43]).

We reported that erbB2 and NF**k**B overexpression play a potential role in CRT resistance and are independently associated with unfavorable survival with marginal significance in MIBC patients treated with induction CRT plus cystectomy (Inoue et al., 2014[17]; Koga et al., 2011[25]). This indicates that erbB2 and NF**k**B are putative therapeutic targets for treatments aimed at improving CRT sensitivity in MIBC. Hsp90 inhibitors at low concentrations, which did not exert cytocidal effects but inactivated erbB2, Akt, and NF**k**B, and efficiently sensitized bladder cancer cells to *in vitr*o and *in vivo* CRT more effectively than sole or combined inhibition of erbB2 and Akt (Yoshida et al., 2011[61]).

Based on these results, we sought to examine the potential role of Hsp90 inhibitors in overcoming the CRT resistance and to encourage clinical trials of Hsp90 inhibitors in patients with MIBC. We are planning a clinical trial of STA-9090 in combination with CRT in patients with MIBC. 

### Combination of Hsp90 inhibitor in cisplatin-based chemotherapy, targeting bladder cancer-initiating cells

Although up to 70 % of patients with advanced bladder cancer show an initially good tumor response to cisplatin (CDDP)-based combination chemotherapy, more than 90 % of patients develop recurrences and eventually die from the disease (Saxman et al., 1997[39]). From the viewpoint of cancer stem cell biology, this phenomenon can be explained as follows: systemic chemotherapy kills the majority of bladder cancer cells, leading to the clinical result of tumor shrinkage; however, a small population of chemo-resistant cancer cells that possess tumorigenic capacity is spared, and they allow tumor regrowth (Dean et al., 2005[4]). The existence of a cellular hierarchy within epithelial tumors has been advocated, and at the top of the hierarchy is a population of tumor-initiating cells (T-ICs) or cancer stem cells. The complete eradication of T-ICs is necessary to ''cure'' advanced cancer patients. 

We isolated bladder cancer-initiating cells (BCICs) from human bladder cancer cell lines based on their CD44 expression status (Tatokoro et al., 2012[51]). These BCICs were more resistant to CDDP and exhibited more activity in the Akt and ERK oncogenic signaling pathways when compared with their CD44- counterparts. Hsp90 inhibitors that simultaneously inactivated both Akt and ERK signaling at noncytocidal concentrations synergistically potentiated the cytotoxic effects of cisplatin on BCICs *in vitro* and successfully sensitized cisplatin-resistant BCIC-derived tumor xenografts to cisplatin. These data encourage clinical trials of Hsp90 inhibitors as they may improve therapeutic outcomes of CDDP-based combination chemotherapy against advanced bladder cancer.

## Future Perspectives

### Hsp90 drug conjugates as novel cancer-specific anticancer agents

An Hsp90 inhibitor drug conjugate (HDC) platform technology was recently advocated (Synta Pharmaceuticals, 2014[49]). HDCs increase cancer cell killing while reducing collateral damage to normal cells. They are small-molecule drugs consisting of an Hsp90 inhibitor (targeting moiety) joined to an anti-cancer agent (payload) via a cleavable chemical linker that is optimized for controlled release of the payload drug inside cancer cells (Ying et al., 2014[59]). The active Hsp90 in tumors acts as a magnet to attract the Hsp90-inhibitor moieties in HDCs, bringing the entire HDC molecule preferentially to tumors. This results in higher concentration and longer duration of the active payload drug inside cancer cells than occurs with standard administration of unconjugated chemotherapy or other payloads. The enhanced delivery creates the potential for greater cancer cell killing and reduced side effects.

HDCs with over 40 different payloads have been developed, including chemotherapeutics, kinase inhibitors, hormone therapies, immunomodulators, and epigenetic modifiers, creating the potential for next-generation compounds in each of these categories (Ying et al., 2014[59]). Examples of payloads include topoisomerase inhibitors (camptothecin), microtubule modulators (taxanes), proteasome inhibitors (carfilzomib), and CDK inhibitors (flavopiridol). This innovative approach could have great potential for reducing the adverse effects of existing chemotherapies as well as overcoming drug resistance mechanisms in multiple human malignancies.

## Figures and Tables

**Table 1:**
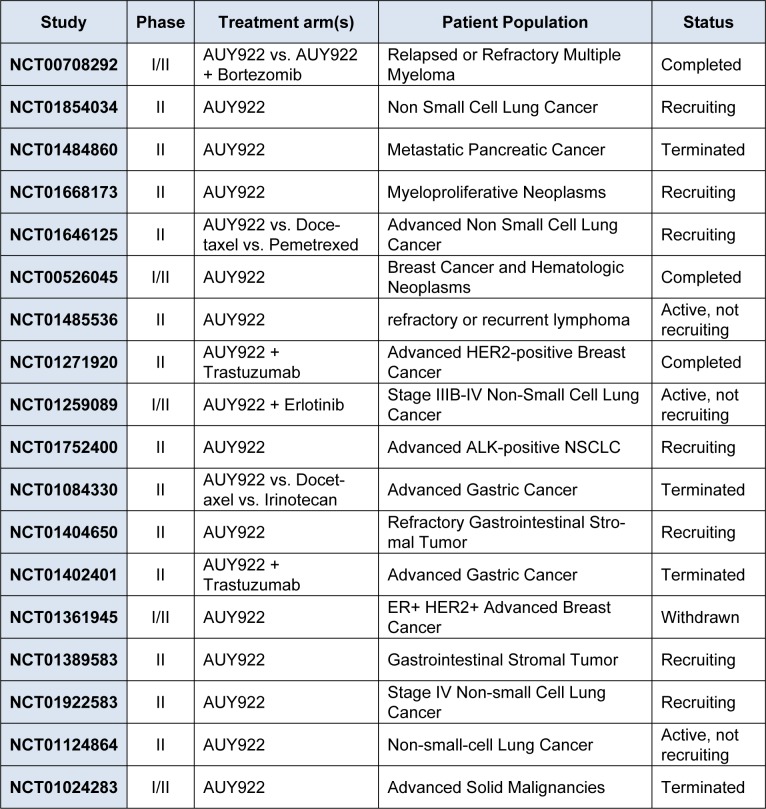
Phase II and III trials of AUY922

**Table 2:**
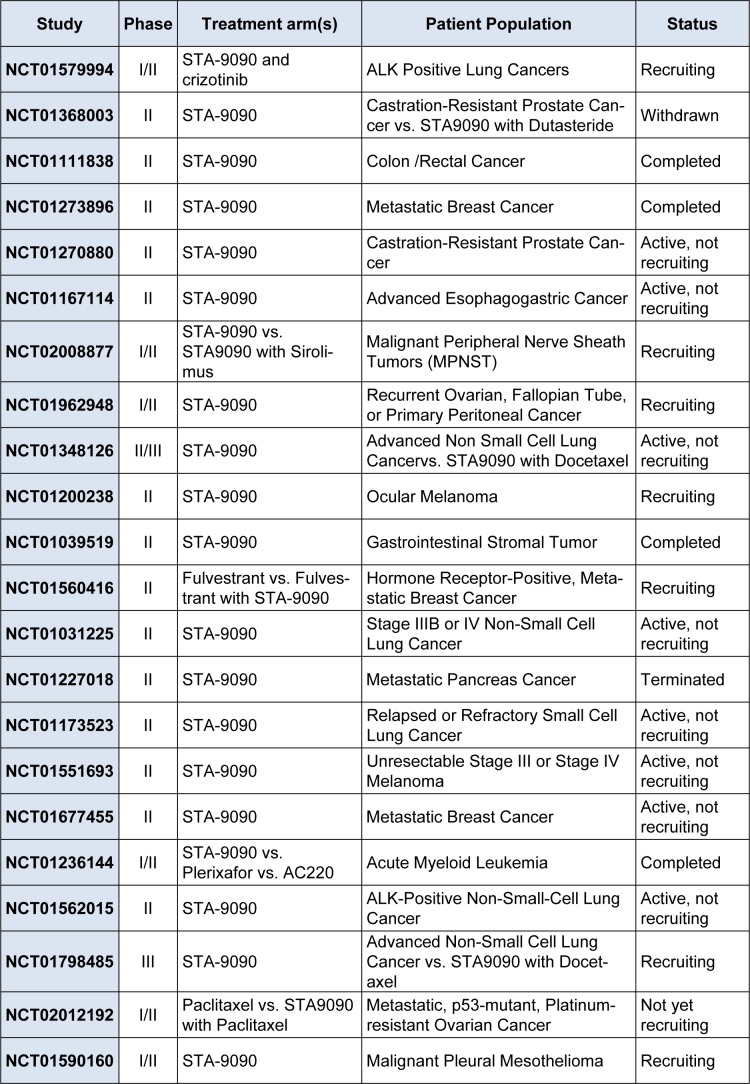
Phase II and III trials of STA-9090
